# The effect of gratitude on death anxiety is fully mediated by optimism in Lebanese adults following the 2023 earthquake

**DOI:** 10.1186/s40359-023-01509-4

**Published:** 2024-01-02

**Authors:** Reem Al Boukhary, Rabih Hallit, Alvaro Postigo, Diana Malaeb, Mariam Dabbous, Fouad Sakr, Sami El Khatib, Feten Fekih-Romdhane, Souheil Hallit, Sahar Obeid

**Affiliations:** 1 School of Medicine and Medical Sciences, Holy Spirit University of Kaslik, Jounieh, P.O. Box 446, Lebanon; 2 Department of Infectious Disease, Bellevue Medical Center, Mansourieh, Lebanon; 3 Department of Infectious Disease, Notre Dame des Secours, University Hospital Center, Byblos, Postal code 3, Lebanon; 4 Department of Psychology, University of Oviedo, Oviedo, Spain; 5 College of Pharmacy, Gulf Medical University, Ajman, United Arab Emirates; 6 School of Pharmacy, Lebanese International University, Beirut, Lebanon; 7 École Doctorale Sciences de la Vie et de la Santé, Université Paris-Est Créteil, Institut Mondor de Recherche Biomédicale, Créteil, France; 8 Department of Biomedical Sciences, School of Arts and Sciences, Lebanese International University, Bekaa, Lebanon; 9 Center for Applied Mathematics and Bioinformatics (CAMB), Gulf University for Science and Technology (GUST), Hawally, Kuwait; 10 The Tunisian Center of Early Intervention in Psychosis, Department of psychiatry “Ibn Omrane”, Razi hospital, 2010 Manouba, Tunisia; 11 Faculty of Medicine of Tunis, Tunis El Manar University, Tunis, Tunisia; 12 Applied Science Research Center, Applied Science Private University, Amman, Jordan; 13 Social and Education Sciences Department, School of Arts and Sciences, Lebanese American University, Jbeil, Lebanon

**Keywords:** Death anxiety, Optimism, Gratitude, Lebanon

## Abstract

**Background:**

Exploring the levels of death anxiety and factors that can undermine its impact are crucial for the Lebanese nationals. Even though studies have shown various relationships between death anxiety and several factors, very few to no research has been done to show the relationship of death anxiety, gratitude and optimism. Therefore, the objectives of our study were to assess the mediating role of optimism in the association between gratitude and death anxiety, along with investigating the validity and reliability of the Arabic version of the Death Anxiety Scale.

**Methods:**

A one-time-point online survey was conducted among Arabic-speaking community adults from the general population of Lebanon (N = 601; mean age 29.91 ± 12.61; 62.7% females). The following scales were used: Scale of Death Anxiety, Optimism–Pessimism Short Scale–2, and Gratitude Questionnaire-Six-Item Form.

**Results:**

The results of the mediation analysis showed that optimism fully mediated the association between gratitude and death anxiety. Higher gratitude was significantly associated with more optimism; higher optimism was significantly associated with less death anxiety. Finally, higher gratitude was not directly associated with death anxiety.

**Conclusion:**

Our study reveals the relationship between gratitude and death anxiety and the mediating role of optimism. Our results need to be confirmed in a longitudinal study, but point to the importance of assessing optimism in prevention and management of persons with death anxiety.

## Introduction

As humans are characterized by self-consciousness, they are destined to live their lives fully aware of the death reality. This awareness can be terrifying for humans, who are wired biologically for self-preservation [1]. Death Anxiety can be classified into two types; (1) Existential Death Anxiety, which includes the issues regarding what results after death, occurs and (2) Tangible death anxiety, which is the anxiety of what occurs to the body in the process and following death.

Death anxiety is recognized as a fundamental fear underlying various mental disorders, encompassing anxiety-related psychopathologies [2], emotional disorders [3], and phobic and compulsive disorders [2]. This fear has the potential to profoundly impact the human psyche, evolving into a terror that deprives individuals of fulfillment and happiness [4]. This existential perspective confirms that death anxiety cuts across a spectrum of psychological disorders [5], emphasizing the importance of addressing it for potential improvements in these conditions. Although various therapeutic approaches have been proposed to alleviate death anxiety (e.g., Yalom’s Existential Psychotherapy [5], Existential–Humanistic therapeutic approaches [6], Cognitive Behavioral Therapy [7] ), its correlates and underlying mechanisms are unclear and not yet well understood. The overall emphasis underscores the significance of addressing death anxiety given its broad impact on mental health and psychopathology. The present study proposes to contribute the literature on this topic by examining the nature and mediator of relationship between gratitude and death anxiety.

### The relationship between gratitude and death anxiety

Gratitude can be defined as “the appreciation and thankfulness for what’s significant and valuable to oneself” [8]. Research consistently showed a negative correlation between death anxiety and gratitude [9, 10], with individuals who exhibit increased gratitude reporting lesser fear of mortality [11]. Notably, gratitude’s positive impact extends to specific populations, as seen in a study involving breast cancer survivors who experienced significantly lower death-related fear of recurrence after undergoing a gratitude intervention [12]. Moreover, gratitude’s benefits go beyond reducing death anxiety and encompass various positive outcomes in interpersonal, psychological, social, and even health-related aspects [13]. Hence, it fosters optimism, promotes support for others, encourages physical exercise, and improves the quality of sleep [13].

Gratitude’s effectiveness in reducing death anxiety can be attributed to several mechanisms. One key mechanism is that when individuals reflect on life events with a thankful attitude, it leads to a profound sense of fulfillment. This sense of fulfillment enables them to perceive their lives as well-lived, subsequently reducing the fear of death [14]. Furthermore, gratitude fosters the discovery of contentment in our current circumstances, regardless of their nature. This, in turn, empowers us to fully embrace the present and willingly accept uncertain outcomes, ultimately reducing the fear associated with both uncertainty and the future. [15]. In a study conducted by Lau & Cheng (2013) [11], researchers investigated the impact of gratitude on death anxiety and positive affect, with a focus on its relationship with optimism, positive self-appraisal, extraversion, and self-esteem. To induce feelings of gratitude, participants in the gratitude group were asked to recollect and write about incidents for which they felt thankful, grateful, or appreciative. The results revealed that the gratitude group reported significantly higher levels of positive affect compared to the hassle and neutral groups. This finding suggests that directing individuals’ attention towards expressions of gratitude in their lives can significantly reduce their fear of death. Furthermore, the study indicates that gratitude may play a role in directing one’s attention towards positive events, contributing to enhanced emotional well-being. That same study [11] proves the need for further investigation of the role of factors involved in positive affect such as optimism and its part in mediation between death anxiety and gratitude.

### Optimism as a mediator in the relationship between gratitude and death anxiety

Optimism refers to a personality trait characterized by the tendency to anticipate the occurrence of pleasant events and to believe in experiencing positive outcomes in the future [16]. Optimism was found to play a role in death anxiety [17]. Higher pessimism is associated with higher death anxiety, while lower death anxiety is linked to optimism [17]. Previous studies have shown an adverse association between optimism and fear of dying [18]. In fact, dispositional optimism has been recognized as a protective factor against death anxiety [19]. Individuals who display high levels of gratitude also tend to demonstrate greater optimism [20]. Gratitude is assessed from multiple perspectives, including making favorable social comparisons, finding joy in modest pleasures, highlighting the positive facets of life, showing appreciation, and recognizing the backing of family and the community. This suggests that these qualities collectively act as robust markers of one’s general sense of well-being. Consequently, individuals who exhibit these traits generally report higher life satisfaction and a greater sense of happiness compared to those with lower levels of gratitude [21]. Although individual studies have explored the correlations and effects of both gratitude and optimism on death anxiety, no research has yet examined the interdependent association between all three factors.

### Rationale

In Lebanon, on February 6, 2023 a massive earthquake took place at around 3am (Beirut Time) with a magnitude of 5.6 followed by another earthquake on February 20, 2023 with a magnitude of 6.3 that shook southern Turkey and hit 256 km north of Beirut, which led so many people to evacuate their houses with huge sensation of tremor and mental stress including anxiety as has happened in the studies of survivors following the 2008 Wenchuan earthquake in China [22], 2016 earthquake of Central Italy [23], 2016 Ecuadorian Earthquake [24], and others. Prior to that, the people of Lebanon have experienced a sequence of highly distressing event such as the 2006 Lebanon War, the COVID-19 Pandemic that greatly impacted the year 2020, the catastrophic explosion at Beirut’s Port, which stands as the largest non-nuclear explosion in history and lastly, the ongoing economic crisis that is jeopardizing fundamental human necessities which all have directly or indirectly related to death anxiety [25]. As heighten levels of death anxiety have been reported in the aftermath of similar traumas, such as war situations [26], influenza A/H1N1 [27], disasters [28], exposure to toxic material [29], we expect that the Lebanese population would also exhibit increased anxiety of death after multiple trauma exposure.

Exploring further the levels of death anxiety and the factors that can undermine its impact are crucial for the Lebanese nationals. Even though studies have shown various relationships between death anxiety and several factors, very few to no research has been done to show the relationship of death anxiety, gratitude and optimism. The objective of the present study was to test the mediating effect of optimism in the relationship between gratitude and death anxiety in a sample of Lebanese adults during the period following the earthquake.

## Methods

### Procedure

Between February and March 2023, a Google Form link was used to collect all data in this cross-sectional study. The study team used the “snowball” strategy, in which they contacted acquaintances and asked them to forward the link to their friends and family. Participation was restricted to adult residents and citizens of Lebanon. Internet protocol (IP) addresses were verified to ensure no one responded to the poll more than once. Following participants’ digital informed consent, the aforementioned items were presented in a pre-randomized order to account for order effects. The survey was voluntarily completed by participants, who received no payment.

### Participants

Six hundred and one Lebanese citizens and residents participated in this study segregated between 224 males and 377 females of a mean age of 29.91 ± 12.61. The participants were majorly single (75.9%), and of different religions and educational levels (Table 1).

### Minimal sample size calculation

A minimal sample of 415 was deemed necessary using the formula suggested by Fritz and MacKinnon [30] to estimate the sample size: $=\frac{L}{f2}+k+1$, where f=0.14 for small effect size, L=7.85 for an α error of 5% and power β = 80%, and k=13 variables to be entered in the model.

### Questionnaire

The questionnaire was written in Arabic, Lebanon’s official language. A digital consent was first obtained, which verified the participants’ willingness to take the survey at their own discretion. A basic description of the study and its instructions were included in the first part. The questionnaire consisted of the following parts:

#### Socio-demographic information

Participants were asked to provide their age, gender, marital status, education level, religion, and residency (flat or house). The Household Crowding Index (HCI) was computed by dividing the total number of inhabitants by the total number of rooms in the household [31]. The socioeconomic status (SES) of the family is reflected by this measure, hence a higher HCI denotes a lower SES. Regarding their financial burden, respondents were asked to answer the question “How much pressure do you feel with regard to your personal financial situation in general?” on a scale from 1 to 10, with 10 referring to overwhelming pressure.

#### Earthquake-related variables

We collected data regarding feeling the earthquake (Yes/No), living a previous disaster (Yes/No), having been injured because of the earthquake (Yes/No), injury/death of a relative, close person or friend in the earthquake (Yes/No), and having been a direct witness of earthquake-related stressors such as collapsed buildings, rescue operations, or the death of earthquake victims (Yes/No).

#### Scale of Death Anxiety (SDA)

The 17-item SDA is a new measure to assess the death anxiety on an individual’s somatic, cognitive, emotional, and behavioral reactions from a symptomatic perspective in Chinese youth samples (e.g. *In the past month, I have often thought of my own death*) [32]. SDA revealed four aspects of death anxiety: Dysphoria, Death Intrusion, Fear of Death, and Avoidance of Death. A 5-point Likert response scale (“1 = Strongly disagree, 2 = Disagree, 3 = Sometimes disagree, sometimes agree, 4 = Agree, 5 = Strongly agree”) was used not only to sensitively distinguish individuals’ responses regarding their feelings and perceptions, but also to reduce their cognitive load and make it easier to respond. The Arabic validated version of the SDA was used [33] (Cronbach’s α in the present sample = 0.97).

#### The Optimism–Pessimism Short Scale–2 (SOP2)^34^

[] measures the psychological disposition of optimism with two items rated on a seven-point Likert scale from not at all optimistic (1) to very optimistic (7) for Item 1 and from not at all pessimistic (1) to very pessimistic (7) for item2. To obtain an optimism scale score, the negatively worded item is recoded, and the unweighted mean score of the two items is computed (e.g. *The next question deals with optimism. Optimists are people who look to the future with confidence and who mostly expect good things to happen. How would you describe yourself? How optimistic are you in general?*). The Arabic validated version was used in this study [35] (Cronbach’s α in the present sample = 0.77).

#### The Gratitude Questionnaire-Six-Item Form (GQ-6)

 Validated in Arabic among Lebanese adults [36], is a six-item self-report questionnaire designed to assess individual differences in the proneness to experience gratitude in daily life (e.g. *I have so much in life to be thankful for*) [37]. Respondents rate each item using a 7-point Likert-like scale ranging from 1 (strongly disagree) to 7 (strongly agree). Higher scores indicate higher gratitude (Cronbach’s α = 0.87).

### Data analysis

The SPSS software v.25 was used for the other statistical analysis. The death anxiety score was considered normally distributed since the skewness ( = − .152) and kurtosis ( = − .027) values varied between − 1 and + 1 [38]. The Student t was used to compare two means and the Pearson test was used to correlate two continuous variables. The mediation analysis was conducted using PROCESS MACRO (an SPSS add-on) v3.4 model 4 (number of replications = 5000) [39]; four pathways derived from this analysis: pathway A from the independent variable to the mediator, pathway B from the mediator to the dependent variable, Pathways C and C’ indicating the total and direct effects from the independent to the dependent variable. Results were adjusted over all variables that showed a *p* < .25 in the bivariate analysis. We considered the mediation analysis to be significant if the Boot Confidence Interval did not pass by zero. *P* < .05 was deemed statistically significant.

## Results

### Sociodemographic and other characteristics of the sample

Six hundred one participants participated in this study, with a mean age of 29.91 ± 12.61 years and 62.7% females. Other descriptive statistics of the sample can be found in Table 1.

**Table 1:** Sociodemographic and other characteristics of the sample (N = 601)

Variable	N (%)
Gender	
Male	224 (37.3%)
Female	377 (62.7%)
Marital status	
Single	456 (75.9%)
Married	145 (24.1%)
Education	
Secondary or les	110 (18.3%)
University	491 (81.7%)
Religion	
Christian	288 (47.9%)
Muslim	313 (52.1%)
Living in a building	
No	106 (17.6%)
Yes	495 (82.4%)
Lived a previous disaster	
No	544 (90.5%)
Yes	57 (9.5%)
Felt the earthquake	
No	46 (7.7%)
Yes	555 (92.3%)
Injury because of the earthquake	
No	600 (9.8%)
Yes	1 (0.2%)
Family or close friend injured because of the earthquake	
No	589 (98.0%)
Yes	12 (2.0%)
Witnessed the consequences of the earthquake	
No	577 (96.0%)
Yes	24 (4.0%)
	**Mean ± SD**
Death anxiety	46.77 ± 14.18
Optimism	4.66 ± 1.27
Gratitude	20.40 ± 4.67
Age (years)	29.91 ± 12.61
Household crowding index (person/room)	1.04 ± 0.51
Financial burden	7.60 ± 2.38

### Bivariate analysis of factors associated with death anxiety

The results of the bivariate analysis of factors associated with death anxiety are summarized in Tables 2 and 3. The results showed that participants who live in a building and those who felt the earthquake compared to not were significantly associated with higher death anxiety. Older age, lower household crowding index and optimism and higher financial burden were significantly associated with more death anxiety.

**Table 2:** Bivariate analysis of factors associated with death anxiety

Variable	Mean ± SD	*T*	*df*	*p*
Gender		− 0.515	599	0.631
Male	46.41 ± 11.73			
Female	46.98 ± 15.46			
Marital status		-1.399	599	0.162
Single	46.31 ± 14.24			
Married	48.20 ± 13.93			
Education		0.577	599	0.564
Secondary or les	47.47 ± 13.22			
University	46.61 ± 14.39			
Religion		1.813	599	0.070
Christian	47.86 ± 1416			
Muslim	45.76 ± 14.14			
Living in a building		-2.100	599	**0.036**
No	44.15 ± 14.16			
Yes	47.33 ± 14.13			
Lived a previous disaster		0.400	599	0.690
No	46.84 ± 14.18			
Yes	46.05 ± 14.20			
Felt the earthquake		-3.532	599	**< 0.001**
No	39.74 ± 14.76			
Yes	47.35 ± 13.98			
Family or close friend injured because of the earthquake		0.385	599	0.707
No	46.81 ± 14.01			
Yes	44.42 ± 21.47			
Witnessed the consequences of the earthquake		− 0.075	599	0.941
No	46.76 ± 13.99			
Yes	47.04 ± 18.49			

Numbers in bold indicate significant *p* values

**Table 3:** Pearson’s pairwise correlations

	Death anxiety	Optimism	Gratitude	Age	Household crowding index
Death anxiety	1				
Optimism	− 0.15***	1			
Gratitude					
Age	0.13**	− 0.04	− 0.01	1	
Household crowding index	− 0.13**	− 0.002	− 0.09*	− 0.24***	1
Financial burden	0.35***	− 0.07	− 0.03	0.26***	− 0.15***

*p < .05; **p < .01; ***p < .001

### Mediation analysis

**Fig. 1:**
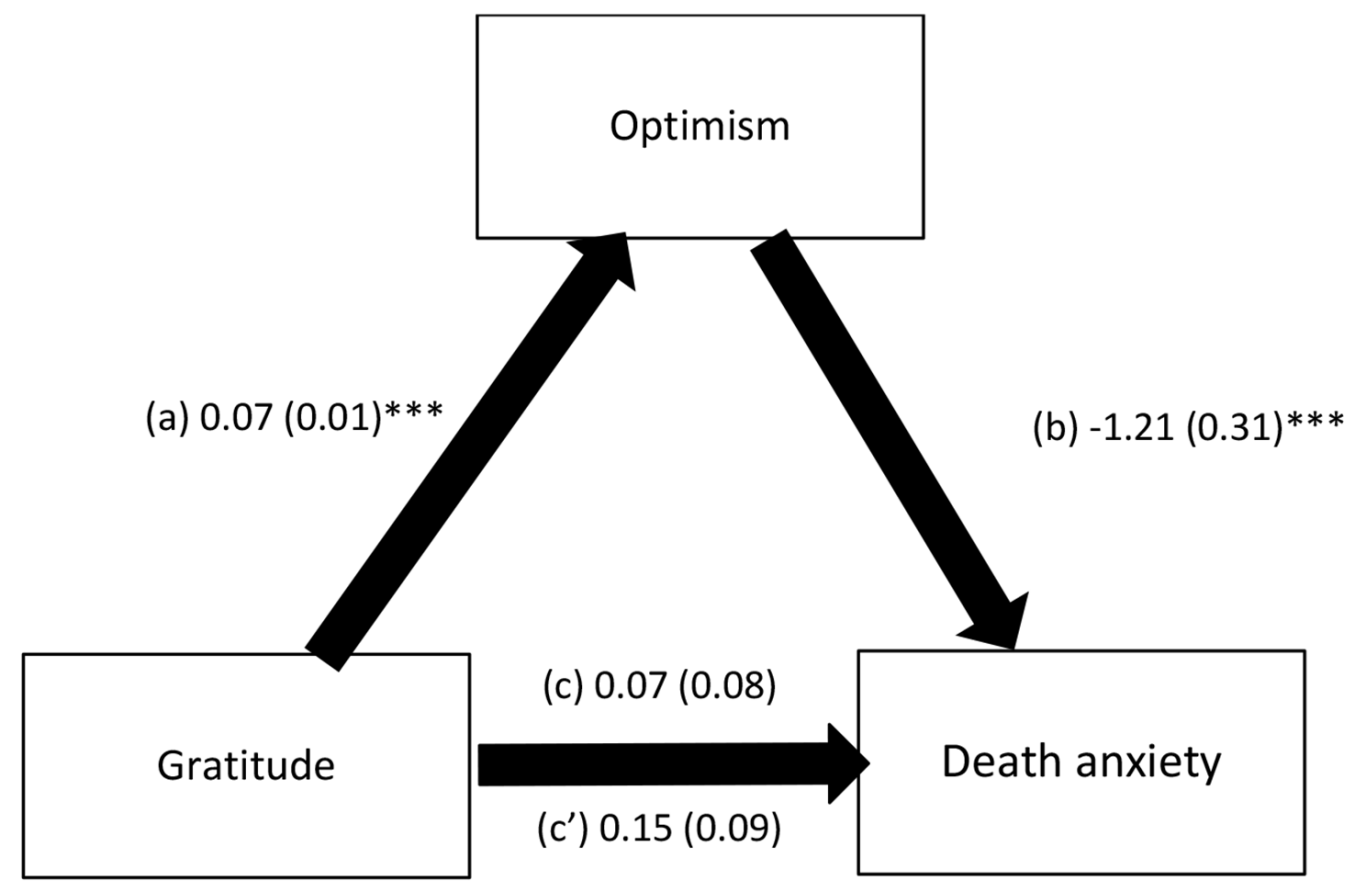
**(a)** Relation between gratitude and optimism (R^2^ = .081); **(b)** Relation between optimism and death anxiety (R^2^ = .162); **(c)** Total effect of gratitude and death anxiety (R^2^ = .141); (c’) Direct effect of gratitude and death anxiety. Numbers are displayed as regression coefficients (standard error). *p < .05; ***p < .001

The mediation analysis was adjusted over the following variables: marital status, living in a building, religion, felt the earthquake, age, household crowding index and financial burden. The results of the mediation analysis showed that optimism fully mediated the association between gratitude and death anxiety (Table 4). Higher gratitude was significantly associated with more optimism (Beta = 0.07, SE = 0.01, *p* < .001, 95% CI 0.05; 0.09); higher optimism was significantly associated with less death anxiety (Beta = -1.21, SE = 0.31, *p* < .001, 95% CI -1.83; − 0.59). Finally, gratitude was not directly associated with death anxiety (Beta = 0.15, SE = 0.09, *p* = .080, 95% CI − 0.02; 0.32) (Fig. 1).

**Table 4:** Mediation analyses results, taking gratitude as the independent variable, optimism and general self-efficacy as mediators and death anxiety as the dependent variable

Mediator	Direct effect			Indirect effect		
	**Beta**	**SE**	***p***	**Beta**	**Boot SE**	**Boot CI**
Optimism	0.15	0.09	0.080	− 0.08	0.03	− 0.14; − 0.03*

*indicates significant mediation. Direct effect refers to the direct association between impact of the gratitude and death anxiety without the effect of the mediator, whereas the indirect effect refers to the same association through the mediator (optimism and general self-efficacy).

## Discussion

Our study unveiled a significant relationship between gratitude and optimism, echoing the findings of previous research. In our investigation, higher levels of gratitude consistently corresponded to elevated levels of optimism. Optimism is a cognitive tool that not only aid in coping with daily negative experiences but also facilitates the perception of such experiences as mildly unpleasant, leaving ample room for recognizing potential positive outcomes on the horizon [40]. Moreover, findings have revealed an expected connection between gratitude and optimism. This association was reported in particular during the COVID-19 pandemic [41]. The observed positive correlation between these traits during times of heightened stress and uncertainty underscores their potential synergistic effects in bolstering psychological resilience and positive outlooks during. A significant contribution of research lies in the examination of the long-term implications of consistent engagement in positive behaviors like expressing optimism and gratitude [42]. The conclusion showed that happiness interventions go beyond mere placebos; their effectiveness is highest when participants are informed about, support, and actively engage in the intervention. This insight holds valuable implications for designing effective well-being programs and interventions [42]. Given the conceptual similarities between optimism and gratitude, it is imperative to acknowledge the studies that have explored their impacts in tandem. Such investigations, comparing and contrasting the effects of these traits, shed light on the distinct yet complementary contributions of each [40]. Also, research highlights the proposition that the effects of trait optimism and trait gratitude are largely autonomous and additive [40].

The current study aligns with prior research [43], where an established negative correlation between optimism and death anxiety levels was identified. Similarly, in another study involving cancer patients [44], it was found that optimism exhibited a negative correlation with the fear of the unknown. This was also supported by the post hoc analyses of a 2018 study by Barnett et al. [17] that revealed a distinct association between pessimism and an increased fear of the unknown. Understanding the intricate relationship between optimism and death anxiety unveils a complex interplay of psychological factors. First and foremost, optimism emerges as a powerful force shaping individuals’ perceptions of the future, fostering cognitive, emotional, and motivational states geared towards positive outcomes [45]. This optimistic perspective leads them to employ coping strategies that prioritize social support and emphasize the positive facets of challenging circumstances. As a result, they exhibit greater mental resilience and adaptability when faced with stressors, which, in turn, indirectly contributes to an improved quality of life [45]. Notably, their optimistic outlook extends to existential concerns such as death anxiety, as they anticipate more favorable outcomes, maintain hope, and exude confidence that things will ultimately go well. This positive mindset equips them to face and conquer negative emotions and frustrations, especially in the face of adversity, such as illness [45]. Furthermore, their propensity for proactive behavior extends to their health, as optimistic individuals tend to be better informed about their well-being and are more likely to engage in preventative measures [46]. Finally, their adaptability is not limited to one coping strategy; rather, they select coping strategies that align with the demands of the specific stressors they encounter [47]. In essence, optimism manifests as a multifaceted trait that not only enhances mental and physical well-being but also influences how individuals approach life’s challenges [47].

In addition to exploring individual associations between gratitude, optimism, and death anxiety, our research ventured into the complex mediation between these three factors. This aspect of our study provides a deeper understanding of how gratitude and optimism may synergistically interact with death anxiety. Findings showed that optimism acted as a full mediator of the relationship between gratitude and death anxiety. A previous study conducted with breast cancer survivors illuminated the constructive impact of gratitude [12]. Those exposed to the gratitude condition demonstrated a proclivity for actively pursuing more desirable goals, a phenomenon likely attributed to the encouragement of death acceptance and the reduction of death-related fears. This observation resonates harmoniously with the *Meaning Management Theory* [48], which posits that when individuals confront their own mortality, they embark on a quest to imbue their lives with significance fostering death acceptance and diminishing death-related anxieties. Findings of previous research also underscore the pivotal role of both gratitude and optimism in predicting enhanced psychological health, including better sleep quality, reduced stress levels, and heightened positive expectations among individuals [40]. As gratitude amplifies the positive facets of daily experiences, while optimism effectively mitigates the impact of negative events [40]. Although both gratitude and optimism share a common emphasis on positive attributes, they differ in their temporal orientations. Gratitude directs attention towards the present and recent past, while optimism propels individuals towards the future [40]. This dynamic interaction sheds light on how individuals perceive and respond to life’s challenges. Understanding these nuanced dynamics is pivotal for designing effective well-being programs and interventions that harness the combined power of gratitude and optimism in regards to death anxiety.

### Clinical implications

Mental health professionals should raise awareness about the importance of gratitude and optimism in maintaining good mental health and alleviating death anxiety. Schools can implement programs to teach students how to develop these skills, and university students can help spread this knowledge.

An important finding of the present study is that the association between gratitude and death anxiety can be fully explained by the mediational mechanism of optimism, meaning that gratitude does not influence death anxiety by itself but by first influencing optimism. This finding has important implications. Clinicians should be aware of the potential protective role of optimism against death anxiety, especially when dealing with victims of trauma. Therapists can utilize the insights provided in this article to assist individuals navigating death anxiety. This involves identifying protective factors, with a particular emphasis on fostering optimism, and recommending interventions to cultivate gratitude. By enhancing gratitude, therapists aim to amplify optimism, seamlessly integrating these strategies into their overall treatment approaches. In addition, although dispositional optimism is often regarded as a trait-like variable [49], there is enough evidence that it can be increased by psychological interventions such as those including the brief in-person Best Possible Self method (For meta-analysis, see [50]).

### Limitations and strengths

Firstly, the cross-sectional nature of our data restricts our ability to establish causal relationships among the three variables under investigation. Furthermore, the use of self-administered questionnaires introduces the potential for information bias, as respondents may occasionally provide random or inaccurate responses due to misunderstandings or misinterpretations of the questions. Additionally, the snowball sampling technique employed in this study raises concerns about selection bias, as it may not fully represent the broader population of interest. Moreover, we must acknowledge the possibility of residual confounding bias. Not all factors associated were considered in our study, and there may be unaccounted variables that could influence the observed associations. However, despite these recognized limitations, our study has yielded intriguing and thought-provoking findings that warrant further investigation within this particular population. These results, while subject to the constraints of our recruitment method, hold potential implications for the broader population. Additionally, it is worth noting that, to the best of our knowledge and based on a comprehensive review of existing literature, no prior studies have explored the mediating role of optimism in the relationship between gratitude and death anxiety. While this presented challenges in directly comparing our findings to previous research, it underscores the significance and originality of our study within the field.

## Conclusion

In conclusion, our study reveals the relationship between gratitude and death anxiety and the full mediating role of optimism. Overall, understanding the roles of gratitude and optimism can aid in developing effective prevention and intervention strategies aiming to improve mental health of traumatized individuals. Subsequent research conducted in different nations may provide valuable comparative insights that could augment the findings of our study. To gain a deeper insight into the dynamic relationships among these three variables, a study focused on causation could be conducted. Additionally, it is crucial not only to elucidate the connection between death anxiety and its influencing factors among Lebanese adults but also to prioritize the development of therapeutic strategies and assess their effectiveness.

## Data Availability

The datasets generated and/or analyzed during the current study are not publicly available due to the restrictions from the ethics committee but are available from the corresponding author on a reasonable request.
